# Short-Term Impact of Oxytetracycline Administration on the Fecal Microbiome, Resistome and Virulome of Grazing Cattle

**DOI:** 10.3390/antibiotics12030470

**Published:** 2023-02-25

**Authors:** Pablo Rovira

**Affiliations:** Instituto Nacional de Investigación Agropecuaria (INIA Uruguay), Treinta y Tres 33000, Uruguay; provira@inia.org.uy

**Keywords:** grazing cattle, oxytetracycline, antibiotic resistance genes, fecal microbiome, virulence

## Abstract

Antimicrobial resistance (AMR) is an important public health concern around the world. Limited information exists about AMR in grasslands-based systems where antibiotics are seldom used in beef cattle. The present study investigated the impacts of oxytetracycline (OTC) on the microbiome, antibiotic resistance genes (ARGs), and virulence factor genes (VFGs) in grazing steers with no previous exposure to antibiotic treatments. Four steers were injected with a single dose of OTC (TREAT), and four steers were kept as control (CONT). The effects of OTC on fecal microbiome, ARGs, and VFGs were assessed for 14 days using 16S rRNA sequencing and shotgun metagenomics. Alpha and beta microbiome diversities were significantly affected by OTC. Following treatment, less than 8% of bacterial genera had differential abundance between CONT and TREAT samples. Seven ARGs conferring resistance to tetracycline (tet32, tet40, tet44, tetO, tetQ, tetW, and tetW/N/W) increased their abundance in the post-TREAT samples compared to CONT samples. In addition, OTC use was associated with the enrichment of macrolide and lincosamide ARGs (mel and lnuC, respectively). The use of OTC had no significant effect on VFGs. In conclusion, OTC induced short-term alterations of the fecal microbiome and enrichment of ARGs in the feces of grazing beef cattle.

## 1. Introduction

Antimicrobial resistance (AMR) is an important public health issue around the world [1,2]. According to recent worldwide estimates provided by Antimicrobial Resistance Collaborators (2022) [3], 1.27 million deaths were attributable to bacterial AMR in 2019. Among the multiple factors involved in the spread of AMR [4], there is concern that the use of antibiotic in cattle could enrich antibiotic resistance genes (ARGs) that confer resistance to antibiotics critically important to human health [5,6]. Therefore, the treatment of cattle with antibiotics is subjected to increased scrutiny because of the potential for public health impacts related to AMR in bacteria that may be transferred from livestock farms to consumer through the food chain or environmental routes [7,8].

Perturbation of the gut microbial community following exposure to antibiotics has been a well-recorded phenomenon in feedlot cattle in North America [8,9,10]. The metaphylactic treatment of entire groups of cattle (‘mass treatment’) is a common practice in feedlot operations, especially in those groups with a higher risk of developing bovine respiratory disease (i.e., newly arrived cattle) [8]. In addition, feedlot cattle usually receive daily in-feed antimicrobials for liver abscess control, which is a hepatic disease caused by invasive bacteria associated with ruminal lesions caused by prolonged high-energy grain-based diets [11,12]. On the other hand, grasslands-based production systems rely upon pastures for feeding animals, with low numbers of animals per hectare, the administration of antibiotics is restricted to individual diseased animals, and no in-feed antibiotics are used [13]. Because of the lack of extensive use of antibiotics, only limited information of the impact of antibiotics on microbial communities is available in grazing beef cattle operations [14].

In extensive systems, tetracycline is the class of antibiotics usually employed to treat infections in animals due to their broad spectrum of activity, high rate of absorption, low toxicity, and low cost [15,16]. However, little is known about the effects of infrequent exposure to tetracyclines on the composition and function of cattle microbial communities. Traditionally, tetracycline resistance in bacterial communities in cattle farms has been investigated using cultures of indicator organisms such as *Escherichia coli* [17], *Salmonella* [18], and *Campylobacter* [19]. This approach offers limited conclusions because it targets specific bacterial isolates and is limited to only a few ARGs [10]. Recently, 16S rRNA amplicon sequencing and shotgun metagenomic approaches have been used to better understand the impact of antimicrobial exposure on entire microbial communities (microbiome) and their associated ARGs (resistome) in food-producing animals [10,20,21]. These and other metagenomic studies have expanded our knowledge of microbial ecology looking at the whole microbiome and the collection of functions they possess [22,23].

In this study, we investigated the impacts of a single dose of oxytetracycline hydrochloride (OTC) on the fecal microbiome, resistome and the collection of virulence factors genes (virulome) in grazing steers using a multi-omic approach. We hypothesized that the injection of OTC will produce significantly short-term changes on the fecal microbiome and will increase ARGs compared to cattle unexposed to OTC, although a gradual return to a similar pre-antibiotic resistome is expected. Increased understanding of the resistome after the sporadic use of OTC in extensive systems will improve our knowledge of AMR ecology aiming to limit ARGs dissemination from cattle to humans.

## 2. Results

### 2.1. Microbiome Diversity and Composition

**Figure 1:**
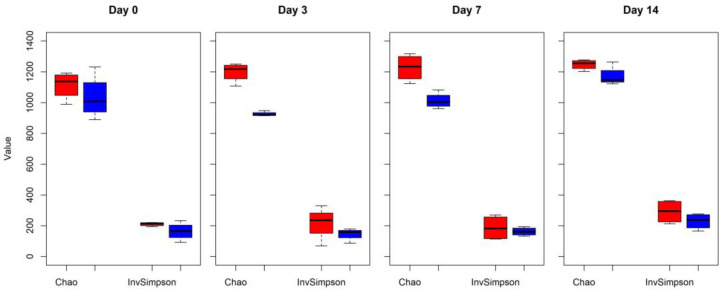
The microbial community alpha richness diversity (Chao and InvSimpson indexes) in cattle feces comparing control (CONT, red) versus antibiotic treatment (TREAT, blue) samples collected on 0, 3, 7, and 14 days.

**Figure 2:**
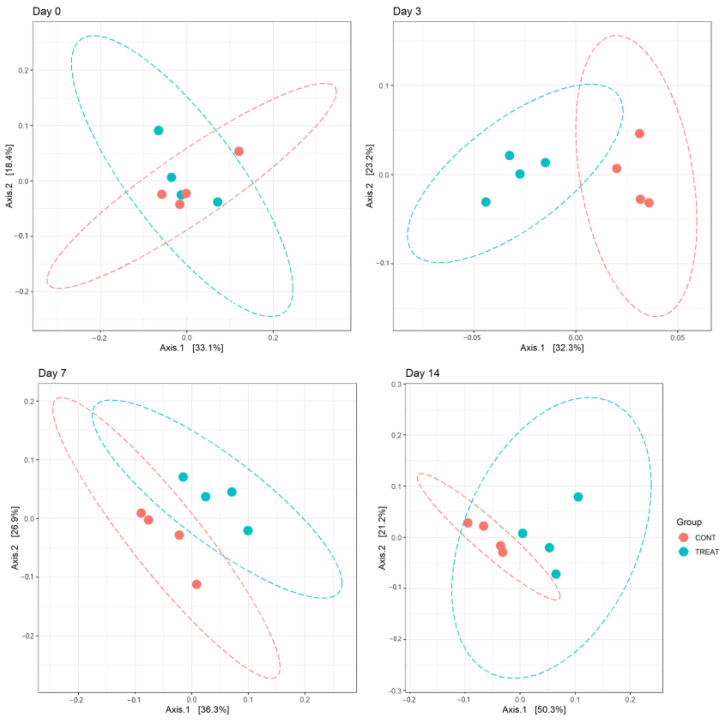
Principal component analysis (PCoA) of the weighted UniFrac distances showing clustering differences (*p* < 0.05) between the microbiomes of control (CONT) and treatment (TREAT) samples collected 3, 7, and 14 days after administration of oxytetracycline hydrochloride to steers.

Changes in alpha diversity indexes Chao and InvSimpson between CONT and TREAT samples are shown in Figure 1. Significant differences were found for Chao (day 3 and 7) and InvSimpson (day 3), with samples in the TREAT group having a decrease in diversity compared to CONT samples. No differences (*p* > 0.05) in Chao and InvSimpson were found between groups on days 0 and 14. Shannon’s diversity index of the TREAT group was significantly lower than the Shannon’s value in the CONT group on day 3 (mean ± s.d.: 5.9 ± 0.1 and 6.3 ± 0.2, respectively), whereas there were no significant differences in Shannon’s diversity between groups on days 0, 7, and 14. No significant differences (*p* > 0.05) between the CONT and TREAT groups were observed for the microbiome beta-diversity on day 0 (Figure 2). However, the fecal microbiome shifted after antibiotic treatment, as shown on days 3, 7, and 14 by a clustering difference (*p* < 0.05) between CONT and TREAT samples for each sampling day post-treatment.

**Figure 3:**
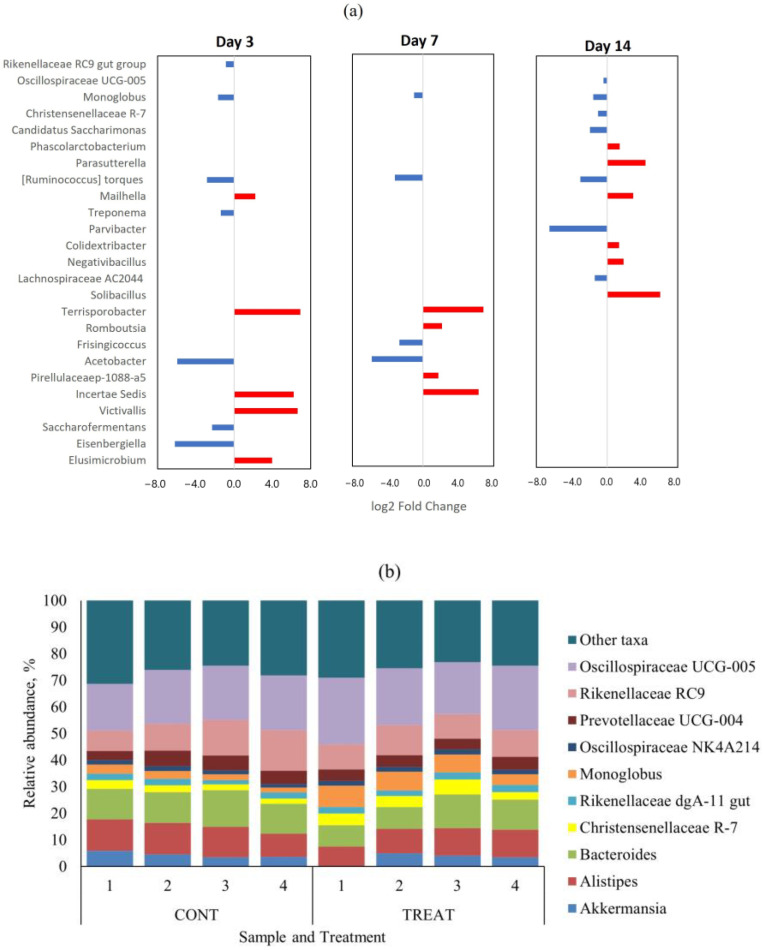
(**a**) Differentially abundant (*p* < 0.05) genera in the fecal microbiome between control (CONT) and oxytetracycline hydrochloride-exposed (TREAT) cattle 3, 7, and 14 days after antibiotic treatment. Taxa in the y-axis are ordered from most abundant (top) to lower abundant (bottom) genera. Negative (blue) and positive (red) log_2_ fold change values within days are genera enrichments in TREAT and CONT samples, respectively. (**b**) Relative abundance of top-10 genera on day 14 separated by treatment.

There were no genera with differential abundance (*p* > 0.05) on day 0 (pre-treatment) between CONT and TREAT samples. The top-10 genera in the microbiome on day 0 were *Oscillospiraceae* UCG-005 (mean relative abundance ± s.d.: 22.1 ± 3.8%), *Bacteroides* (10.7 ± 1.4%), *Rikenellaceae* RC9 (10.2 ± 3.6%), *Alistipes* (6.9 ± 1.1%), *Monoglobus* (6.8 ± 2.6%), *Prevotellaceae* UCG-004 (5.7 ± 0.8%), *Christensenellaceae* R-7 (5.1 ± 1.5%), *Candidatus Saccharimonas* (3.9 ± 1.4%), *Akkermansia* (2.8 ± 1.3%), and *Prevotellaceae* UCG-003 (2.5 ± 4.0%). Those genera represented 79.2 ± 4.0% of the fecal microbiome on day 0 across treatments. Differentially abundant taxa at the genus level that were significantly different (*p* < 0.05) between CONT and TREAT samples on days 3, 7, and 14 are shown in Figure 3a. Among the 159 genera found on day 3, 12 (7.5%) were differentially abundant (*p* < 0.05) between CONT and TREAT samples. Similarly, 8/158 (5.1%) and 13/165 (7.9%) genera were differentially abundant (*p* < 0.05) on days 7 and 14, respectively, in CONT and TREAT samples. In general, low abundant taxa were more affected on days 3 and 7 after antibiotic administration, except for more abundant *Rikenellaceae* RC9 and *Monoglobus* genera that were enriched in TREAT samples. Figure 3b shows the relative abundance of top-10 genera on day 14 separated by treatment. *Oscillospiraceae* UCG-005, *Monoglobus*, *Christensenellaceae* R-7, and *Candidatus Saccharimonas* ended up the experimental period on day 14 being enriched (*p* < 0.05) in TREAT samples compared to CONT samples.

### 2.2. Antibiotic Resistance Genes

**Figure 4:**
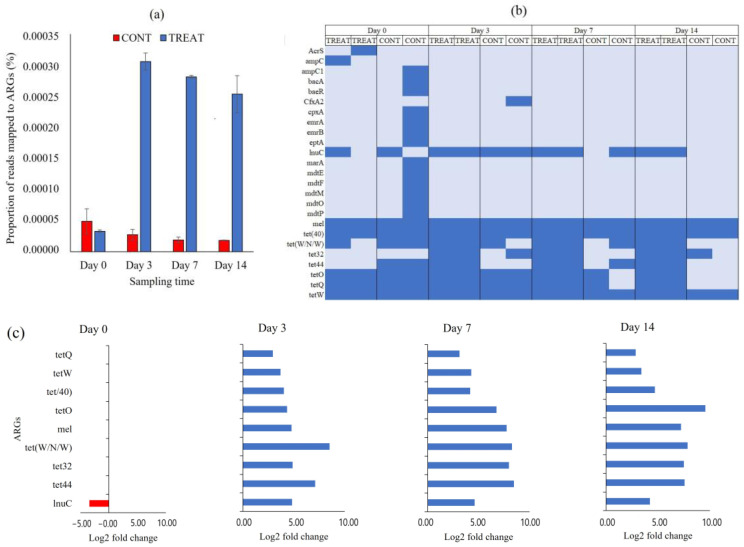
(**a**) The proportion of short sequencing reads mapped to antibiotic resistance genes (ARGs) in control (CONT) and oxytetracycline-treated (TREAT) cattle in fecal samples on days 0, 3, 7, and 14 following oxytetracycline administration. (**b**) Heatmap of absence (light blue) and presence (blue) of ARGs between CONT and TREAT samples throughout the experimental period. (**c**) Differentially abundant (*p* < 0.05) ARGs in the fecal resistome between CONT and TREAT cattle 0, 3, 7, and 14 days after antibiotic treatment. ARGs in the y-axis are ordered from most abundant (top) to lower abundant (bottom) genes. Negative and positive log_2_ fold change values are ARGs enrichments in CONT (red) and TREAT (blue) samples, respectively.

We quantified the burden of ARGs in CONT and TREAT cattle groups using a metagenomic approach. The proportion of short metagenomic reads mapped to ARGs in the CARD database, as a proxy of AMR burden, significantly increased in the cattle that received an OTC injection, and the difference was maintained until the end of the experimental period (Figure 4a). Cattle that received the OTC treatment had between 11 and 14 times greater AMR hits than cattle in the CONT group. After the assembling of short reads and identification of ORF, a total of 25 ARGs were annotated conferring resistance to tetracycline (*n* = 7; tet32, tet40, tet44, tetO, tetQ, tetW, tetW/N/W), fluoroquinolone (*n* = 2, emrA and emrB), peptide (*n* = 2; bacA and eptA), cephalosporine/penam (*n* = 2; ampC and ampC1), aminocoumarin/aminoglycoside (*n* = 2; cpxA and baeR), acridine dye/nucleoside (*n* = 2; mdtP and mdtO), lincosamide (*n* = 1; lnuC), cephamycine (*n* = 1; CfxA2), and to more than two classes of antibiotics (*n* = 6; mel, AcrS, mdtE, mdtF, mdtM, marA) (Table S1). From the 25 ARGs detected, almost two-thirds (16/25) were only found in one or two samples (Figure 4b). Moreover, 13 of those 16 ARGs were recovered from the same CONT sample on day 0, and they were no longer detected during the rest of the experimental period. The core resistome was composed by nine ARGs present in >50% of samples (lnuC, mel, and all tet genes). Samples collected from TREAT cattle showed greater ARGs richness (*p* < 0.05) than samples collected from CONT cattle on days 70 and 14. In those sampling times, feces from TREAT cattle had greater Shannon’s resistome diversity index (*p* < 0.05), which accounts for both the abundance and evenness of ARGs per sample. Shannon index averaged 1.93 (TREAT) and 1.15 (CONT) on day 7 and 1.86 (TREAT) and 1.00 (CONT) on day 14.

Only those ARGs present in the core resistome were considered for differential abundance statistical analysis. On day 0, when none of the animals had been exposed to antibiotic, only lnuC was significantly different between CONT and TREAT groups (Figure 4c). Following OTC treatment, all individual ARGs in the core resistome were more abundant (*p* < 0.05) in TREAT samples than in CONT samples on days 3, 7, and 14 after antibiotic exposure.

### 2.3. Virulence Factor Genes

**Figure 5:**
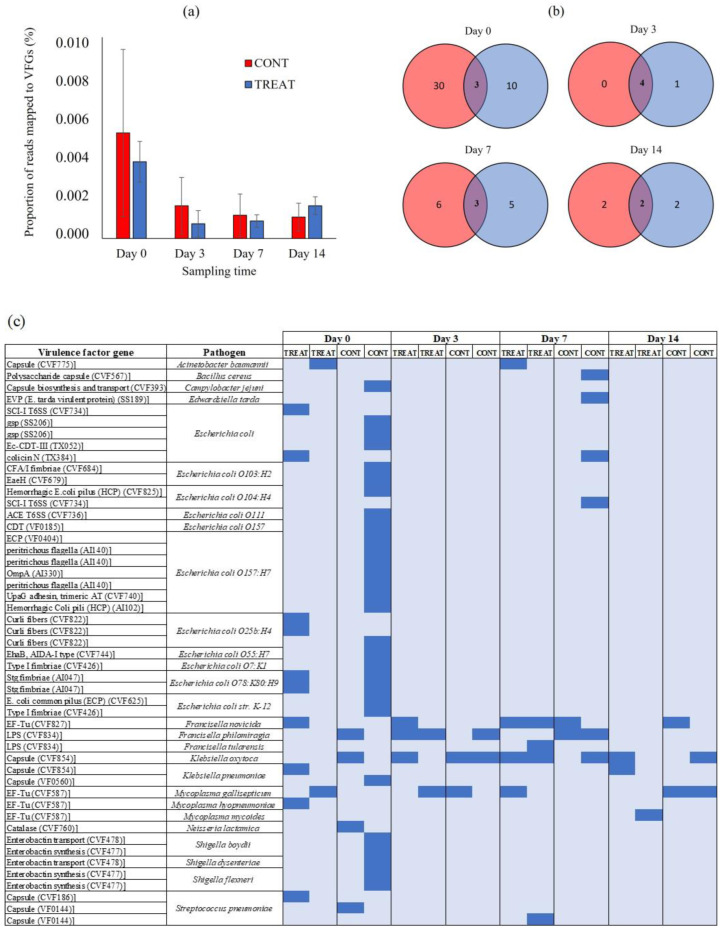
(**a**) The proportion of short metagenomic reads mapped to virulence factors genes (VFGs) in fecal samples of control (CONT) and oxytetracycline-treated (TREAT) cattle on days 0, 3, 7, and 14. (**b**) Venn diagram showing the unique and shared VFGs between CONT and TREAT groups on each sampling time. (**c**) Heatmap of absence (light blue) and presence (blue) of VFGs between CONT and TREAT samples throughout the experimental period.

There were no differences (*p* > 0.05) in the proportion of short metagenomic reads mapped to VFGs in the VF database between CONT and TREAT samples (Figure 5A). On day 0, 0.005% (CONT) and 0.004% (TREAT) of the short metagenomic reads mapped to VFGs in the VF database. The proportion of mapped reads did not increase (*p* > 0.05) on the following sampling times, averaging 0.001% (CONT) and 0.002% (TREAT) on day 14. After assembling the short metagenomic reads, a total of 49 VFGs were detected corresponding to 11 pathogens: *Escherichia coli* (*n* = 27), *Shigella* spp. (*n* = 5), *Streptococcus pneumoniae* (*n* = 3), *Francisella* spp. (*n* = 3), *Mycoplasma* spp. (*n* = 3), *Klebsiella* spp. (*n* = 3), *Acinetobacter baumannii* (*n* = 1), *Bacillus cereus* (*n* = 1), *Campylobacter jejuni* (*n* = 1), *Edwardsiella tarda* (*n* = 1), and *Neisseria lactamica* (*n* = 1) (Table S2). There were 43 VFGs found on day 0 showing a great natural variation among samples (mean: 12; min.: 2, max.: 31) (Figure 5B). Interestingly, the sample in the CONT group on day 0 that had the highest number of ARGs also had the highest number of VFGs. Across all samples and sampling times, the most prevalent VFGs were associated with *Klebsiella* spp. (69% of samples), *Francisella* spp. (62%), and *Mycoplasma* spp. (50%) (Figure 5C). Only three VFGs were unique to TREAT animals following antibiotic administration: a defensive virulence factor capsule of *Streptococcus pneumoniae* (cps4I) found 7 days post-TREAT, an essential enzyme (glucose-1-phosphate thymidylyltransferase) mediating the virulence and adherence of *Francisella tularensis* to host tissue found 7 days post-TREAT, and a membrane-associated translation elongation factor (tuf) on *Mycoplasma mycoides* found 14 days post-TREAT.

## 3. Discussion

Although antibiotics have been investigated for their effect on the fecal microbiome and resistome in feedlot cattle [8,9,24], there is little information on their effect on pasture-based systems of production where cattle usually have less exposure to antibiotics [14]. In these systems, individual animals sporadically receive short-term antibiotic treatment if prescribed by an authorized veterinarian, and therefore, it is important to know the potential collateral consequences on the bovine microbiome and resistome, which are not usually exposed to the selective pressure exerted by antibiotics. Using complementary approaches based on the analysis of 16S rRNA amplicon and shotgun metagenomic sequencing, we conducted the first controlled study in Uruguay to evaluate the fecal microbiome and its resistome among cattle exposed to a single dose of OTC. We observed that the microbiome diversity of treated cattle was significantly disturbed in the short term, increasing ARGs conferring resistance to tetracyclines compared to control animals without exposure to OTC. This finding is consistent with previous studies that reported the perturbation of the gut microbiome following antibiotic treatment within a few days after antibiotic exposure [25,26,27].

The greatest effect of OTC on the fecal microbiome richness was observed on day 3 after antibiotic administration based on alpha diversity measures in CONT and TREAT groups (Figure 1). This was not unexpected given that was the most immediate sampling time following treatment and likely when antibiotic concentrations were highest [14]. Xia et al. [28] reported that the plasma concentration of OTC peaked 6–8 h after intramuscular injection of the drug, but active plasma concentrations were present for ~60 h. Although urine is the main route of excretion of OTC, fecal excretion can reach 40% [28] suggesting a close contact between the drug and the microbial population present in bovine feces. As a result of such contact, the fecal microbiome of cattle was characterized by reduced Chao, InvSimpson, and Shannon values 3 days after OTC administration. Loss of bacterial community richness as a result of antibiotic treatment has been previously observed in studies of humans [29] and in the bovine rumen and feces [19,30]. Since alpha diversity measures are frequently used as an indicator of microbiome ‘health’ with high values associated with a stable and healthy gut microbial environment [31,32], the return to the values of alpha diversity observed prior to the effect of the antibiotic would be desirable to achieve.

Beta diversity results of weighted PCoA plots indicated the cluster separation of the CONT and TREAT groups on days 3, 7, and 14 after OTC injection (Figure 2). While microbiome alpha diversity differences between CONT and TREAT groups disappeared 14 days after OTC exposure, beta diversity was still different between both groups. This could be explained because weighted beta diversity takes into account the relative abundance of the different taxonomic groups in addition to presence or absence. The major driver of the differences between CONT and TREAT microbiomes on day 14 were genera assigned to *Clostridia*, which were still enriched in TREAT samples 14 days after OTC exposure (*Oscillospiraceae, Monoglobus*, *Candidatus Saccharimonas*, *Ruminococcus torques*, *Lachnospiraceae*) (Figure 3). The observation that particular genera increased in abundance may be due to opportunistic bacteria (i.e., antibiotic-resistant or unreached by the antibiotic) that flourish when microbiomes are disrupted [33]. However, as we did not quantify the absolute abundance of taxa (i.e., spiked samples), we were unable to determine the exact relationship between affected taxa comparing CONT and TREAT samples. Overall, these results are consistent with previous studies involving antibiotics in healthy individuals [34,35], which support the hypothesis that antibiotics cause a significant dysbiosis in the microbiome. However, the 14-day study window of our study limited the ability to discern whether the observed alterations to the taxonomy and microbiome diversity persisted to longer intervals. As a result, further work should include follow-up beyond 2 weeks to determine the enduring impact of OTC on gut microbiome.

Although cattle enrolled in the present study had not been previously treated with antibiotics, we found ARGs in feces from all cattle on day 0. This finding was consistent with data from other studies [13,36,37] and supports the concept that bacterial AMR is a naturally occurring phenomenon that would exist with or without the use of antibiotics [38,39]. Further, tetracycline resistance has been reported in animals and environments which apparently are not under the direct exposure of antibiotic contamination such as national parks and wild animals [40,41]. However, any antibiotic use may increase the occurrence of AMR by increasing the mobilization and dissemination of AMR among microbial communities through horizontal gene transfer and/or clonal expansion of resistant taxa [5,42,43]. In our study, we found 25 unique ARGs conferring resistance to 11 several classes of antibiotics (Figure 4b). If we exclude one sample which was very diverse and complex in terms of its resistome, the most common ARGs conferred resistance to tetracycline, macrolides, streptogramin, and lincosamide. Other studies performed in intensive beef cattle production systems (feedlots) using similar shotgun metagenomic approach and sequencing depth found >100 unique ARGs [8,44]. It suggests that cattle raised in extensive systems with low exposure to antibiotics may harbor lower amounts of ARGs and less diverse resistome than cattle raised in confined animal feeding operations daily exposed to in-feed antimicrobials.

In the present study, the administration of OTC was associated with a greater amount of metagenomic reads aligned to ARGs in the CARD database (Figure 4a). Although the magnitude of the difference between CONT and TREAT groups was maintained until 14 days after OTC exposure, there was a downward trend in the proportion of mapped ARGs in TREAT samples from days 3 to 7 (−8%) and from days 7 to 14 (−10%). This suggests a slow tendency of returning to pre-treatment AMR levels in the TREAT group following OTC exposure. An abundance of tetracycline-resistance genes, including tetW, tet40, tetQ, tetO, tetW/N/W, tet32, and tet44, was higher in feces of cattle injected with OTC compared to cattle not given OTC (Figure 4c). Those genes provide resistance through ribosome protection protein (RPP), which is considered the second most important mechanism of the tetracycline resistance in bacteria, after tetracycline efflux pumps, and it was originally attributed to Gram-positive bacteria, but nowadays, they are often found in a variety of Gram-negative species [15,45]. Santamaría et al. [13] and Yang et al. [41] reported that RPP was the more common mechanism of resistance found in tetracycline resistance genes in beef cattle operations, whereas other studies reported that tet genes encoding efflux pumps were more frequently found in dairy cattle and diverse agricultural environments [46,47]. The most prevalent RPP genes found in this study (tetO, tetQ and tetW) are potentially transferred between bacteria as they are associated with mobile genetic elements such as plasmids, conjugative transposons, and integrons [45,47]. Quantification of the burden, mechanisms of resistance, and localization in bacterial genomes of tet genes in farm animals can be used as a baseline to evaluate the variation of the AMR level over time and to establish associations of the use of antibiotics in animal production systems and spread of AMR [48,49].

The association between antibiotic usage and homologue resistance (i.e., tetracycline use and tetracycline ARGs) has been demonstrated before in cattle [50,51,52]. In our study, OTC usage was also associated with increased ARGs conferring resistance to macrolides (mel) and lincosamide (lnuC). The mel gene is necessary for erythromycin resistance and is predicted to interact with other macrolide resistance genes such as mefE and mefA [53]. Furthermore, the most abundant tetracycline resistance gene found in the present study, tetQ, is associated with conjugative transposons in *Bacteroides* spp. which often carry erythromycin resistance genes [54]. The lnuC gene is a transposon-mediated nucleotidyltransferase conferring resistance to lincomycin by converting the antibiotic to a nontoxic form [55]. The fact that mel and lnuC ARGs increased, even though macrolides and lincosamide were not used in this study, suggested that ARGs can be selected and enriched without using the respective antimicrobial because of their co-localization in complex resistance clusters sharing mechanisms of resistance [56,57,58]. Knowledge about co-resistance can contribute to target intervention strategies to reduce unintended enrichment and the spread of emerging genes conferring resistance to critically important antibiotic agents. It is also essential to determine which critical ARGs should be including for monitoring during surveillance [49,59].

The abundance of VFGs was analyzed using the VF database (Figure 5). Overall, 88% (43/49) of the VFGs detected during the study were already present on day 0 prior to antibiotic administration. This result suggests that intestinal bacteria in cattle is a natural reservoir of VFGs [60,61]. The similar burden of VFGs detected in CONT and TREAT cattle on days 3, 7, and 14 suggests that antibiotic treatment was unlikely to be a practice promoting VFGs dissemination through pathogen enrichment in the gastrointestinal tract of cattle. As the entire host’s microbiome is under antibiotics’ selective pressure, not only bacterial pathogens but also the association between ARGs and pathogenic VFGs weakens [62]. Moreover, the increase in the number of reads aligned to ARGs conferring resistance to tetracyclines observed after antibiotic administration in TREAT cattle did not correlate with an increase in VFGs, suggesting that those tetracycline-resistance genes were enriched in commensal bacteria that may act as a reservoir of ARGs [51,63]. Across all samples, VFGs associated with *Klebsiella* spp., *Francisella* spp., and *Mycoplasma* spp. were the most detected, having been present in 11 (68%), 10 (62%), and 8 (50%) out of the total samples, respectively. These pathogens have been previously isolated in cattle and rural environments [64,65,66], but they are not usually the most common pathogens found in the gastrointestinal tract of beef cattle, suggesting the possibility of horizontal VFGs transfer to other bacterial groups. On the other hand, VFGs associated with *Escherichia coli* and *Shigella* spp. were associated with few animals on day 0.

To our knowledge, this study provides the first insight into the development of AMR and pathogenic VF following the administration of OTC in grazing cattle in Uruguay using a multi-omics approach. This approach is advantageous in that it enables a broad identification of ARGs present in the resistome [67]. However, the fact that we identified 25 unique ARGs in cattle feces did not mean that they were fully expressed conferring phenotypic resistance. For that reason, Forslund et al. [68] proposed the term ‘resistance potential’ instead of ‘resistance’ to reflect potential differences in ARGs expression and regulation that can affect phenotypic resistance. In addition, the findings presented here are limited to a single group of animals in a single farm, which may limit the extrapolation to wider populations. Given the observed individual heterogeneity on ARGs carriage on day 0, it is possible more comprehensive sampling would have been necessary to have greater robustness in the results. Despite the limitations of the present study, the results suggest avoiding the indiscriminate use of antibiotics (mass treatments) or without a clinical diagnostic (‘just in case’ prescription) would help to slow the rise of AMR in livestock. This targeted approach would reduce the frequency of the peaks of ARGs fecal excretion observed after antibiotic treatment. In addition, it will reduce the cost associated with antibiotic use without compromising productivity in grazing systems and will improve the perception of beef cattle producers in a society concerned about the dissemination of AMR in public health.

## 4. Materials and Methods

**Figure 6:**
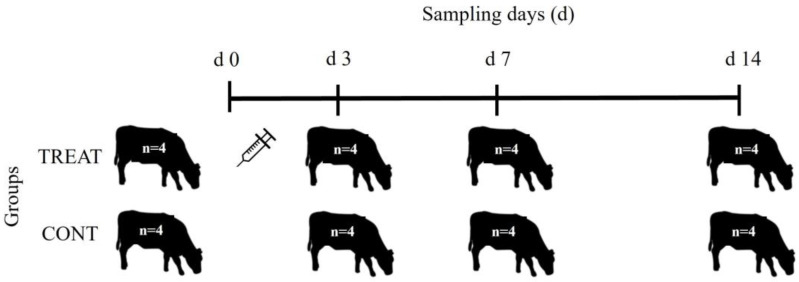
Experimental design and timeline for fecal sampling. TREAT: treatment group that received a single intramuscular injection of 1 mL of oxytetracycline hydrochloride (200 mg) and sodium diclofenac (5 mg) suspension (Oximic Plus LA, Microsules, Uruguay) per 10 kg body weight on day 0.

Eight yearling (12 months old) Aberdeen Angus steers were randomly selected from a larger herd to participate in the study. These animals had not received any antibiotic treatment from birth. They were randomly assigned to one of two groups (*n* = 4 animals per group): control (CONT) and treatment (TREAT). On day 0, individual fresh fecal samples were obtained from the rectum using a new palpation sleeve for each animal (Figure 6). Following initial sampling, cattle in the TREAT group received a single intramuscular injection of 1 mL of oxytetracycline hydrochloride (200 mg) and sodium diclofenac (5 mg) suspension (Oximic Plus LA, Microsules, Uruguay) per 10 kg body weight. After treatment, CONT and TREAT groups were kept separately freely grazing native grasslands. The stocking rate was 0.8 steers/ha corresponding to a paddock of 5 ha for each group of cattle. The two paddocks were physically separated by ~400 m. All cattle were then sampled at days 3, 7, and 14 following the same procedure than day 0. All fecal samples (*n* = 32) were immediately placed on ice and then stored at −20 °C until DNA extraction.

Samples were transported refrigerated to the Laboratorio Tecnológico del Uruguay (LATU, Montevideo, Uruguay) and genomic DNA was extracted using the SureFood^®^ Advanced kit (R-Biopharm AG, Darmstadt, Germany) according to manufacturer’s instructions. The concentration of DNA was measured using the Qubit dsDNA HS assay kit (Invitrogen^TM^, Waltham, MA, USA). Final eluted DNA (70 µL aliquots) from individual samples were sent to the Macrogen Inc. Laboratory (Seoul, South Korea) for library preparation and sequencing. Individual 16S rRNA amplicon libraries (*n* = 32) were constructed using Macrogen’s default primers Bakt_341F: CCTACGGGNGGCWGCAG and Bakt_805R: GACTACHVGGGTATCTAAT targeting 16S (V3-V4) region. Amplicons were sequenced on an Illumina MiSeq platform (Illumina Inc., San Diego, CA, USA) generating 300 bp paired-end (PE) reads. Shotgun metagenomic sequencing was performed in two pooled samples per group for each sampling time, totaling 16 samples. Metagenome libraries were generated using the Illumina TruSeq DNA protocol (350 bp insert size) and were sequenced on a NovaSeq6000 instrument (Illumina Inc., CA, USA) generating 150 bp PE reads.

Amplicon 16S rRNA sequences were processed with DADA2 [69] to quality filter, trim, denoise, merge the PE reads, and infer amplicon sequence variants (ASVs). After the removal of chimeric sequences, the obtained ASVs were compared to the SILVA database v138.1 [70] for taxonomic classification. The DECIPHER package [71] was used for the alignment of multiple ASVs sequences, and a phylogenetic tree was built using the phanghorn package [72]. The phyloseq package [73] was used to combine sample metadata, ASVs table, phylogeny and taxonomic assignment objects into a single phyloseq object. To evaluate the alpha diversity of bacterial communities in CONT and TREAT groups, we compared the community richness and diversity indices Chao, Shannon, and InvSimpson. Those indices were estimated after rarefying each group of samples to the sample with the least number of sequences. Principal component analysis (PCA) was determined to evaluate the microbiome similarity between CONT and TREAT groups (beta-diversity).

Metagenomic PE raw reads were trimmed using Trimmomatic version 0.36 [74]. Trimmed reads were de novo assembled using MEGAHIT v1.1.2 [75] keeping contigs with length ≥ 500 bp for downstream analysis. The contigs were subjected to the prediction of open reading frames (ORF) using PRODIGAL [76] using the default minimum gene length (90 nt). Using Diamond [77] v0.9.30.131 (with parameter: e value < 10–5, >90% identity at the protein level, and >80% query sequence coverage), gene sequences were searched against the databases CARD v3.1.2 [78] and VFDB [79] for annotation of ARGs and virulence factor genes (VFGs), respectively. In addition, trimmed short reads were aligned using Barrows–Wheeler Alignment (BWA) version 0.6.2 [80] to ARGs contained in the same CARD database to quantify their abundance. Only individual ARGs that were covered >80% in length by sample reads and present in more than two samples were considered for downstream analysis. For each ARG in each sample, the total number of aligned reads was summed to create a count matrix with samples in columns and ARGs in rows.

Alpha diversity indexes were statistically analyzed using the non-parametric Kruskal–Wallis test, and significant differences between groups were determined using the Wilcoxon Rank Sum test. Significant differences in microbiome composition were evaluated using permutational multivariate analysis of variance (PERMANOVA) in the Vegan package in R [81]. Analysis of differentially abundant taxa among the treatments at the genus and ASV level were determined using the package DESeq2 based on the negative binomial distribution method [82]. After mapping the metagenomics reads to the CARD database, the resulting ARGs count table was normalized using a cumulative sum scaling (CSS) method, log2 transformed, and analyzed for differential abundance using a zero-inflated Gaussian distribution model contained in the metagenomeSeq R package [83]. The analysis of similarity (ANOSIM) included in the vegan package was used to test similarities between the groups being compared.

## 5. Conclusions

In summary, ARGs and VFGs were detected from all fecal samples collected from cattle before antibiotic exposure. Given the lack of previous antibiotic treatments in the animals enrolled in this study, we confirmed that cattle are natural carriers of ARGs. The selective pressure exerted by OTC following its administration altered the composition of the microbial community and promoted enrichment of tetracycline-resistant genes in treated cattle up to 14 days after exposure. Therefore, even a single course of antibiotics commonly used for the treatment of bacterial infections in cattle can have prolonged effects on ARGs levels in fecal microbiomes. However, those changes in the microbiome and resistome were not linked to an increase in VFGs in treated cattle. The results of this study have important implications, recommending the prudent use of antibiotics even in farms with a low frequency of treatments to prevent the sporadic proliferation of critically important ARGs.

## Data Availability

Raw sequencing data obtained from genomic DNA in the present study are publicly available on January 2024 or upon publication (whichever is first) at the National Center for Biotechnology Information (NCBI) database with Bio Project accession numbers PRJNA911263 (16S rRNA) and PRJNA 911298 (shotgun metagenome).

## Supplementary Materials

The following supporting information can be downloaded at: https://www.mdpi.com/article/10.3390/antibiotics12030470/s1, Table S1: Description of antibiotic resistance genes detected after mapping assembled metagenomes to the CARD database; Table S2: Description of virulence factors detected after mapping assembled metagenomes to the virulence factor database (VFDB).
